# Potential effect of different nutritional labels on food choices among mothers: a study protocol

**DOI:** 10.1186/s12889-020-8411-8

**Published:** 2020-03-06

**Authors:** Shirin Seyedhamzeh, Saharnaz Nedjat, Hedayat Hosseini, Elham Shakibazedeh, Anthony J. Viera, Ahmadreza Dorosty Motlagh

**Affiliations:** 1 Department of Community Nutrition [DCN], School of Nutritional Sciences and Dietetics [SNSD], Tehran University of Medical Sciences [TUMS], Tehran, Iran; 2 Students’ Scientific Research Center, TUMS, Tehran, Iran; 3 School of Public Health [SPH], TUMS, Tehran, Iran; 4 Department of Food Sciences & Technology, National Nutrition &Food Technology Research Institute, Shahid Beheshti University of Medical Sciences, Tehran, Iran; 5 Department of Health Education and Promotion, SPH, TUMS, Tehran, Iran; 6 Department of Community and Family Medicine, Duke University, Durham, NC USA; 7 DCN, SNSD, TUMS, No 44, Hojjat-dost Alley, Naderi Street, Keshavarz Boulevard, Tehran, 1416-643931 Iran

**Keywords:** Nutrition labeling, Food labeling, Physical activity

## Abstract

**Background:**

The prevalence of non-communicable diseases (NCDs) is increasing in the world. Healthy food choice and adequate physical activity are key factors in preventing NCDs. Food labeling is a strategy that can inform consumers to choose healthier foods at the point of purchase. In this study, we intend to examine the status of existing labels and to clarify their strengths and weaknesses. Then, for the first time in Iran, we will design a type of physical activity equivalent calorie label and will test it on some food groups of packaged products including dairy products, sweetened beverages, cakes, and biscuits.

**Methods:**

This study will be conducted in two phases. In phase 1, nutrition fact labels and traffic light labels will be assessed through focus group discussions and interviews among different groups of mothers, industrialists and nutrition and food industry specialists as to determine strengths and weaknesses of the current labels on packaged products. Then, the initial layout of the physical activity calorie equivalent label will be drawn with respect to the viewpoints received from mothers. Thereafter, we will include the scientific opinions to it for creating the first draft of our new label. In phase 2, a total of 500 mothers of students 6–12 years old randomly assigned to five groups. The study groups will be as follows: (1) without nutrition label group, (2) current traffic light label group, (3) current traffic light label group in which, a brochure will be used to inform mothers, (4) physical activity calorie equivalent label group, and (5) physical activity calorie equivalent label group in which a brochure will be used to inform mothers. Some samples of dairy products, beverages, cakes, and biscuits will be presented. ANOVA and multiple linear regressions will be used to examine the association between the label type and the main consequence (energy of the selected products) and secondary outcome (time).

**Discussion:**

The effect of the new food labels will be evaluated based on the differences between the calories of selected food groups.

**Trial registeration:**

Iranian Registery of Clinical Trials [IRCT]20,181,002,041,201 N1.

## Background

The global prevalence of non-communicable diseases (NCDs) is increasing, particularly in low- and middle-income countries [1]. The World Health Organization estimated that 39% of adults aged ≥18 years were overweight and 13% were obese in 2016 [2].

According to the available scientific evidence [3–5], major NCDs, other than road injuries can be reduced through healthy nutrition. In addition, long-term strategic policy-making can reduce the increasing trend and excessive costs of NCDs [3].

Food environment policies can affect all people, because they have influence on behavior and can make a great part of daily choices [6]. In this regard, environmental changes and policy-making should be accompanied by programs that motivate people and enable them to choose healthier foods and improve their physical activity [7]. Globally, there are various policies to modify people’s eating behaviors. Such policies include food labeling [7–9], taxes and subsidies [9], and marketing and quality standards [10, 11].

Food and meal labeling have been established with the aim of influencing consumers’ choices [12]. The effectiveness of food labels has been studied over various durations [13, 14] through randomized [15, 16] and non-randomized [17, 18] interventions. Most studies in this regard have been carried out in high-income countries such as North America and Europe [19, 20].

In Iran, various labels including nutrition information have been used for over 10 years. Starting from 2014, traffic light labeling on food products had been optional by the Food and Drug Administration of Iran, but it became mandatory for all packaged products after 2 years [21]. The results of a review of the data from United States and Canada suggest the use of a traffic light on packaged products might be helpful [22]. The effectiveness of such labels has not been checked in Iran yet [23].

One of the newer types of labeling recently introduced shows the amount of physical activity including distance or time needed to burn off the calories in a food item [24]. Based on the data of three focused group discussions (FGDs) data, Dowray et al. designed physical activity calorie equivalent labels for the consumer and tested them in a hypothetical scenario study. In that cross-sectional study, calorie content of the selected fast food meals was defined as the outcome. Two types of physical activity label were designed that had presented the amount of miles or minutes needed to burn off calories. A label displaying physical activity in distance had the greatest influence. However, most participants preferred a label expressing physical activity in minutes required to burn calories as opposed to distance. At the end, 82% of the subjects preferred a physical activity calorie equivalent label [24]. In the study by Antonelli et al. 80% of participants believed that a physical activity calorie equivalent label could affect their dietary choices and also increased their motivation for physical activity [25].

Therefore, this study will be conducted to investigate the status of packaged products in Iran, including traffic light food label and nutrition information label, to identify their strengths and weaknesses. Another important objective of current study is to design a physical activity calorie equivalent label considering views of consumers, experts, and executives for the first time in Iran. Then, the new physical activity equivalent calorie label will be compared with current traffic light food label of packaged products. To achieve this, the products will be presented with either the new designed physical activity label or the current traffic light label to determine the effects of these labels on food choices.

## Methods and design

This study will be conducted in two phases.

### Phase 1

#### Step I: assessing of existing labels on packaged products

For step I, during a thematic analysis, all types of labeling covering nutritional information and traffic light labels will be assessed through FGDs and interviews with different groups of mothers, nutrition and food industry specialists, and industrialists. To gather mothers’ opinions from five different regions (North, South, East, West, and Central), the current map of 22 municipal districts of Tehran will be used. First, five districts out of 22 will be randomly and then two elementary schools (6–12 years) will be selected from each of those 5 districts (a total of 10 elementary schools). Using the available list of schools in the Education Department of selected regions simple randomization. Thus, 10 elementary schools will be chosen and at least ten FGDs will be held in those selected schools. To invite participants, the mothers will be contacted by phone up to when 6 of them accept to co-operate. Homogeneous purposeful sampling regarding education level will be used [26]. On the day that FGD will be conducted, the investigator will introduce herself and the note taker as well as the aim of this study. Then consent form will be signed by mothers and a demographic questionnaire will be completed by face to face interview. All FGDs will be recorded by a voice recorder and a note taker takes all the discussions during the sessions. Each session will consist of two parts: in the first part, the Iranian existing “traffic light label” and “nutrition fact label” will be discussed in order to clarify the strengths and weaknesses. The Iranian Ministry of Health has duty to check the accuracy of current labels provided by factories and we trust those information. Thus we will not try to check such information in our current study.

In the second part, the concept of a physical activity calorie equivalent label will be introduced and we will ask the participants for their opinions about it, and necessary information will be obtained. The length of these sessions will be 90 min.

After FGDs conducted with mothers, while summarizing the FGDs findings, at least six semi-interviews will be conducted with quality managers and industry experts from different groups of dairy products, cakes, biscuits and sweetened beverages. Before interviews, a semi-structured questionnaire will be designed by the investigator. The purposeful snowball sampling will be applied [26]. In this regard, subjects from famous brands will be interviewed and the interviewees will recruit other experts according to the aim of the study. Before conducting the interviews, the interviewees will be aware of the purpose of the research and the interviews will be recorded by a voice recorder after asking for their permission. Furthermore, participants will be assured that the information would be used only for research purposes and would not be accessible for others who are not in the research team.

As the final activity of step I, the results of mothers’ FGDs and industrialists’ interviews will be presented to a group of nutritionists and food industry experts in separated FGDs through a short 10-min presentation. In FGDs with nutritionists and food industry experts, the strengths and weaknesses of traffic light label and nutrition fact label will be identified, the expectations of mothers regarding the physical activity calorie equivalent label will be discussed, and the experts’ comments will be collected.

#### Step II: designing the new label based on physical activity calorie equivalent

In step II, the initial layout of the physical activity label will be drawn by a graphic artist with respect to the viewpoints of the mothers. The label will be changed by consensus among researchers based on the mothers’ and experts’ opinions. Ultimately, the final version. of physical activity calorie equivalent label will be designed.

As step II, the initial layout of the physical activity label will be drawn by a graphic artist with respect to the viewpoints received from all 3 groups. Thereafter, the first draft of the Physical Activity designed label will be emailed to mothers participated in FGDs asking for their opinions. The label will be changed by consensus among researchers based on mothers and experts’ opinions. Ultimately, the last version of physical activity calorie equivalent label will be designed.

### Phase 2: intervention

Similar to phase 1, 10 elementary schools will randomly be selected from five districts of Tehran. The mothers of children from these schools will be recruited to assess the effect of the new label. To invite the participants, the mothers will be contacted by phone, until 50 literate mothers accept to cooperate. After obtaining informed consent, demographic data will be collected and mothers will be asked to complete the International Physical Activity Questionnaire (IPA) [27] in order to determine whether they have any especial interest in physical activity that may affect their attention to physical activity calorie equivalent labeling. Five hundred mothers will be randomly assigned to enter the study and each 100 one of them will go to one of the five groups. No relevant information regarding use of food labels will be given to mothers, except for two groups. The study groups will be as follows: (1) without nutrition label group, (2) current traffic light label group, (3) physical activity calorie equivalent label group, (4) current traffic light label group with a brochure for more information, and (5) physical activity calorie equivalent label group with a brochure for more information. A “food choice” brochure will be handed to mothers describing the labeling information in detail and the participants will be asked to read it after filling their questionnaires.

We will present products of each groups in one classroom and so, we will have 3 classrooms for participants. The first classroom will include food items “without nutrition label”, the second classroom will be for “current traffic light label” and “current traffic light label group with a brochure for more information” and finally the last classroom will be presented by foods with “physical activity calorie equivalent label” and “physical activity calorie equivalent label group with a brochure”. Milk, yogurt and cheese will be in the dairy group. Due to different brands designs, there will be at least 30 dairy products. Twenty beverages and twenty cakes and biscuits will be shown in other rows. All the foods in five food groups will be similar in brand and type.

Products included in the “without nutrition label” group will provide no nutrition information. There will be no change in the traffic light label group and the labels will appear exactly as produced by factories. Calories and serving sizes of products on labels will be based on factories definition and we do not change them. Each particular food with either no label, or old traffic light label, or our newly designed label will be presented into 3 classrooms similarly. Groups in traffic light label and physical activity calorie equivalent label will only be different in the form of figures and presentation of some information such as physical activity. Finally, the products with physical activity calorie equivalent labels will be covered with the new designed label. Each mother will be asked to imagine she is in a supermarket, choose one product that she wants from each category to buy for her family; place them in a bag inside the room, and then leave there. One of the researchers will record food items. For example, one package of creamy cake has about 320 cal, while there is another cake with only 130 cal; so we expect mothers to choose better option based on the information provided by the labels. Thus, at the end of the study, the total calories from each category as well as the total calories of all selected foods will be compared among different 5 groups. For each of the five groups, the “time” spent by each participant while selecting food items will be recorded by the researcher. After the food choice stage, a series of short questions will be asked in order to assess the mothers’ attention to the mentioned labels. All the collected data will be entered into a database.

Accordingly, the major consequence obtained in this step will show the effect of the new designed label that focuses on the differences in calories for each food group. Accordingly, the major consequence obtains from this step would be the effect of the new label on choosing lower calories goods by consumers and the second outcome would be the time spent while food selection according to different labels.

#### Randomization method in phase 2

Stratified balanced blocked randomization will be applied. Strata are schools, and each of the five blocks consists of ten participants in every school. The blocks and sequence within block will be generated using the Stata Statistical Software and will be given to the person who will manage random allocation of individuals in the school. This method will balance the number of participants in all groups and ensure allocation concealment. The assessor and the analyzer will be blind to groups’ randomization and outcome evaluation.

**Fig. 1:**
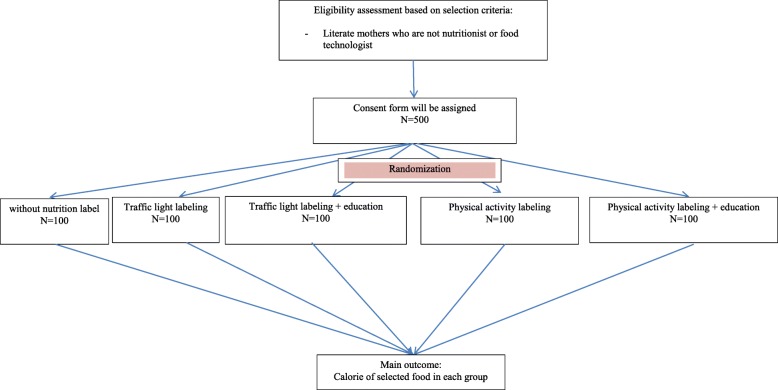
participants’ flow diagram (phase 2)

**Fig. 2:**
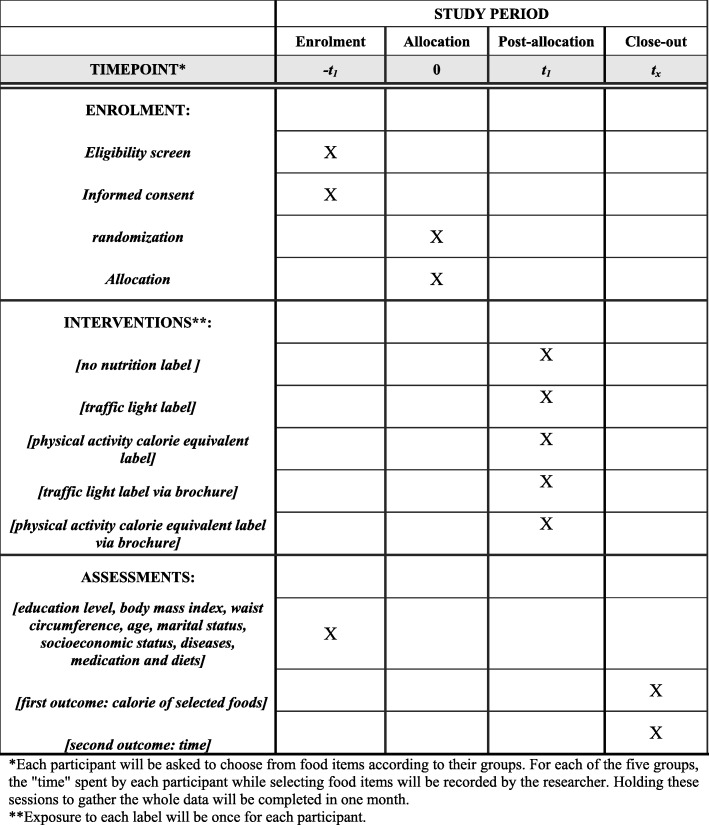
The schedule of enrolment, interventions, and assessments of participants in phase 2

Mothers will be selected by a person other than the investigator. On the other hand, mothers will select food products while they are alone in the room. The diagram of the intervention phase and the time schedule are shown in Figs. 1 and 2, respectively.

### Selection criteria

Participants will be literate mothers who are willing to participate in the study. The mothers who are nutritionist or food technologist will not be included.The age of students will be 6–12 years. However, we will put no limitation for age of mothers who will enroll the study.

### Phase 1

FGDs will consist of 6 to 10 people and at least 6 industrialists and experts will be interview, and will continue until data saturation is achieved.

### Phase 2

#### Method of calculating sample size and number

We calculated the number of participants by using G*Power software 3.1 [28]. In this regard power analysis was applied based on a linear multiple regressions with an assumed small to medium effect size (f = 0.18). The total sample size in this case will be 481 with power of 0.9. The sample size is 96 in each arm after attrition, and we scaled that up to *n* = 100 therefore, 500 subjects will be studied in total. Due to the multiple comparisons, alpha level will be adjusted and interpretation of statistically significant results will be done by taking conservative cautions.

#### Qualitative analysis of data in phase 1

The MAXQDA 10 software will be used to analyze the interviews. This software is used to analyze social-oriented texts [29]. Qualitative analysis with thematic approach will be used. It will be done as follow. First, data will be analyzed after each group discussion or interview; for this purpose, all the data will be entered into the software in a textual format. The strengths, weaknesses, description, and information for the new physical activity calorie equivalent label will be defined as categories, and each category will be stored in a special part of the software. Thereafter, decision will be made on coding units after reading the text for several times and the codes will be added to each relevant category at the same page of the software. At this point, classification will be done using the data of the study, and the stability of the coding will be tested. The coding stability will be re-checked after encoding the full text in the software. Finally, the structures of meanings will be provided based on the data inference.

#### Statistical analysis of data in phase 2

Analysis of Variance (ANOVA) will be used to examine the relationship between the label type and the main consequence showing calorie of the selected products and the second outcome which is spent time during food selection. The purpose of this analysis is to see whether the difference between different groups has a significant effect on the main outcome. In order to adjust the effect of confounders (education level, body mass index, age, and socioeconomic status) multiple linear regressions will be used and the energy of the selected food products in each group will be analyzed. In all tests, type I error will be considered 5% and the data will be analyzed using the Stata 12 and SPSS version 16.

### Ethical considerations

This study is approved by Tehran University of Medical Sciences (96–03–161-37,037). It has also been registered in the Iranian Registry of Clinical Trials (IRCT20181002041201N1).

In the qualitative section, the participants will be informed of the study objectives.

Before conducting the interviews, the interviewees will be briefed of the purpose of the research and participants will be assured that the information would be used only for research purposes and would not be accessible for others who are not in the research team. In addition, no sensitive date will be requested and hence, no such data will be provided. The information of all participants will remain confidential and caution will be made to avoid referring to the names or terms that could reveal the identity of the individual while implementing the interviews. First author will take consent letter from participants and they will exclude from the study whenever they wish to.

Mothers with 18.5 > BMI > =25 and children with − 1 < height Z-score < + 1 and/or − 1 < BMI Z-score < + 1 will be considered as under and/or over-nourish and will be provided with free counseling and training by a registered dietitian if they wish to.

In the quantitative phase, the participants will be asked to choose from different food groups while they are unaware of the purpose of the study. In similar studies, to examine the actual effect of the intervention, the participants are not informed about the reason for the intervention [30, 31] and they are unaware of the main purpose. In addition, the participants’ information will remain confidential and their contribution will be acknowledged.

### Limitations

The final stage of the study will be simulating the environment since the mothers are actually in the sample food selection room, which is one of the study limitations. However, for the purpose of examining the effect of labels, there will be a comparison group and since the conditions are the same for all groups, it will largely moderate the conditions. Furthermore, the mothers will be blinded to the main goal of intervention.

Another limitation is assigning the selection process only to mothers and not involving fathers in the study, so that gender differences will not be clear. The reason for targeting the mothers is that they are more involved in purchasing and preparing food and in different studies [32, 33] it has been founded that women use food labels more than men. Due to Iranian previous studies [34, 35] the use of nutrition labels among people was rare. Thus, we decided to consider women who might be more responsible for preparing foods in households.

In the present study health literacy or nutrition knowledge will not be measured whilst nutritionist or food technologist mothers will not enter in our study. Previous studies in Iran did not report any significant variances of knowledge or usage of nutrition labels [34–36].

## Discussion

Physical activity calorie equivalent labeling is in the testing phase and since this type of labels focuses on the amount of energy received, it can encourage individuals to choose low calorie foods [31]. However, such studies in the real world are scarce [30, 31, 37] and have been mostly done in high-income countries [24, 25, 31, 38, 39]. A combination of training and physical activity calorie equivalent labels can be an effective intervention [29].

A recent systematic review and meta-analysis compared the effect of calorie labeling and physical activity calorie equivalent labeling on calorie ordering of foods in restaurants. The results of this study showed no significant difference in the amount of calories ordered between these two types of labels, although the amount of calories ordered in real condition studies were 65 cal lower than studies in unreal conditions [40].

To the best of the authors’ knowledge, this is the first comparison of physical activity calorie equivalent labels with traffic light labels. The results of this study can be used by policy-makers in three scenarios: 1- collecting comprehensive views of mothers as household representatives, industrialists as executive representatives, and nutritionists and food industry experts as scientific representatives about the existing labels, 2- modifying the current labels according to the results of this study, 3. presenting a new label that meets the needs of consumers.

## Data Availability

Not applicable.

## Abbreviations

FGD: Focus Group Discussion
IPAQ: International Physical Activity Questionnaire
NCD: Non Communicable Disease
